# Supplementary material for: Are carbon emissions trading and green financial instruments synergistic? -Comprehensive quantitative research based on content analysis
Source: PLoS One. 2024 Mar 7;19(3):e0298601. doi: 10.1371/journal.pone.0298601 (PMC10919601; doi:10.1371/journal.pone.0298601)
Supplement: S1 Data — (ZIP) [file pone.0298601.s001.zip › Data/地级市Z_F工作报告.zip!/░▓╗╒/═¡┴Ω╩╨/└┤╫╘╣1⁄2╓┌║┼í╛┬φ┐╦╩2╛▌═°í┐_2013.docx]

各位代表： 

现在，我代表市人民政府，向大会报告工作，请予审议，并请市政协委员和其他列席人员提出意见和建议。 

一、2012年和过去五年工作回顾 

刚刚过去的2012年，全市人民在省委、省政府和中共铜陵市委的坚强领导下，认真贯彻落实科学发展观，积极应对外部经济环境的挑战，凝聚力量，务实进取，实现了经济平稳增长、社会和谐稳定，为“四个之城”建设奠定了良好的基础。 

预计，全市生产总值增长10%。财政收入增长10.1%，其中地方财政收入增长40.2%。全社会固定资产投资增长28%。社会消费品零售总额增长16%。城镇居民人均可支配收入增长13.2%；农民人均纯收入增长17.5%。居民消费价格指数2.5%。新增就业3万人。完成节能减排目标。 

一年来，我们重点抓了以下工作： 

第一，促进经济平稳增长。加大招商引资工作力度，实际利用外资4.9亿美元，增长23%；实际利用内资570亿元，增长54%。实施“项目推进月”行动，全年在建亿元以上项目295个，新开工亿元以上项目55个。有色公司“双闪”、铜化大合成氨等重大项目建成投产。实施“抓调控机遇”行动，资源型城市转型试点再获国家五年政策支持，全年争取国家和省转移支付资金27.8亿元，增长21%。实施“保经济增长”行动，百名干部深入企业、帮扶企业，累计减免税费和发放专项支持资金17亿元。有色公司主营业务收入突破千亿元，铜化集团突破百亿元，全威铜业、精达铜材跻身“中国民营企业五百强”。 

第二，推进产业转型升级。制订并实施六大战略性新兴产业规划，把铜基新材料作为首位产业来抓，战略性新兴产业产值保持全省第3位。“圈区管理”获国家批准建设，国家铜铅锌质检中心列入国家支持项目。完成“智慧铜陵”建设总体规划。中科院皖江产业中心正式运行。扎实推进国家质量强市示范市创建工作。严格环评、能评，倒逼传统企业实施节能改造和减排项目。实行服务业发展目标责任制管理，落实服务业重点项目投资总额324亿元。金园港埠码头建成使用，江南文化园三期、台湾城、青铜帝国一期等项目开工建设。 

第三，统筹城乡规划建设。按照全域规划理念，完成新一轮城市总体规划、土地利用总体规划、城乡一体化建设规划和近期建设规划修编，完成部分重点区域城市设计。强力推进以“惠民、便民、育民、亲民”为重点的文明城市创建活动，城市文明程度指数测评居全国地级城市第13名。长江二路等主干道路建成使用，长江路地下人防工程快速推进。铜陵长江公铁大桥和宁安城际铁路铜陵段建设顺利。西湖新区路网框架全面拉开。东部城区、南部城区基础设施逐步完善。经济技术开发区循环化改造启动。产业转移示范区起步区骨干路网基本贯通。美好乡村启动建设，完成166个自然村危房改造和村庄整治，成功申报国家现代农业示范区。 

第四，深化经济体制改革。城乡一体化改革向纵深发展，发放流动人口居住证2.3万张，完成74万户籍人口户别标注，率先实现城乡养老、低保制度并轨；出台农村产权改革“1+8”方案，农民宅基地和农房登记确权发证工作进入常态化，农村产权抵押融资进入试点阶段。大建设体制逐步完善，修订了政府投资项目管理办法、实施细则和责任追究办法；加强了政府投融资平台建设；完成市、区财政体制和部分领域事权关系调整。深化行政管理体制改革，开展专项资金、事业单位绩效考核，启动政府部门履职情况社会化评估；试点财政专项资金资本化运作；完善行政审批制度改革，推行首接办结制，引入行政审批中介服务，开通行政审批电子监察系统；扩大政府购买社会组织服务试点范围。交通运输等现代服务业“营改增”稳步实施。事业单位分类改革平稳推进。社区综合体制改革深度拓展，以社区为平台的各类公共服务、志愿服务更加贴近群众。成为全省首个全国城镇发展改革试点市。 

第五，提高居民生活品质。继续把改善生态环境质量放在突出位置，淘汰落后产能企业40户，全面关闭可采区之外的石料矿山，基本实现城关滨江化工区整体关转；启动滨江生态岸线整治工程、十万亩森林增长工程以及废弃矿山植被恢复首批项目；严格超标排放等行为监管，全年空气质量一级天数由上年的23天增加到43天。精心实施民生工程，全年投入11亿元，完成48项民生工程，群众受益90%以上；新开工保障性住房12039套、竣工10804套，保障房建设和管理的经验在全省推广；率先实施重特大疾病医疗救助制度；实施城乡高龄津贴制度。企业退休职工养老金标准提高到1435元，城乡低保标准提高到355元。 

第六，加快社会事业发展。制订了教育现代化建设方案。在全省率先试行二级以上医疗机构“先看病后付费”服务，在社区开展家庭医生服务。公共文化场所全部免费开放。成功举办第十二届中国（铜陵）青铜文化博览会、省第三届体育大会。全国幸福家庭试点市、全民健身示范试点市、无障碍城市和国家创新型城市创建工作扎实开展。实现全国双拥模范城“三连冠”，成为全国慈善百强城市。妇女儿童、红十字、档案、史志、民族、宗教、外事、侨务、对台、外宣、消防、民防、气象等各项事业取得新进步。扎实推进“法律七进”等活动。60个社会管理创新项目顺利实施，重大事项社会风险评估、“四大四进四送”等制度常态化，信访工作实现“四个零”的目标。安全生产形势总体平稳。 

在推进经济社会发展的同时，我们切实加强政府自身建设。自觉接受人大法律监督和政协民主监督，密切与工会、共青团、妇联等人民团体的联系。认真执行人大决议，按时办结人大代表建议124件、政协提案273件。深入开展廉政风险防控工作，加强审计监督和效能监察。大力推进政务公开，率先实行部门预算和审计结果公开，开通政府微博集群“铜陵发布”，实行网络问政，自觉接受人民群众监督。 

各位代表！过去一年的工作为本届政府画上了圆满句号。回首五年，我们经受住国际金融危机持续加深的各种风险挑战，在科学发展实践中创造了辉煌业绩，超额完成市十四届人大一次会议确定的任务，总体实现提前八年全面建成小康社会的目标。 

五年来，综合经济实力大幅提升。全市生产总值由2007年的279亿元增加到610亿元，财政收入由50亿元增加到127亿元，分别增加1.2倍和1.5倍。人均生产总值和财政收入分别达到83000元和17000元，是2007年的2.1倍和2.5倍。累计完成固定资产投资1748亿元，是2007年之前历年投资总和的3.2倍。 

五年来，城乡建设取得重要成就。城市框架基本拉开，建成区面积由46平方公里扩大到64平方公里，城市化率达到75%。争取京福高铁在铜陵过江，区内“六纵六横”干线路网基本建成。100%的村通公路、通宽带、通广播电视，93%的村通客运班车，95%的农户喝上安全饮用水，76%的农户用上卫生厕所。 

五年来，改革开放实现重大突破。社区综合体制改革成为全国典型。大部制、新农合和城乡一体化改革走在全国前列。率先完成基层医药卫生体制和文化体制改革。公车改革在全国率先推开。累计实际利用内、外资1500亿元和16亿美元，是前五年的8倍和4.3倍。 

五年来，生态环境有了明显改善。万元GDP能耗下降21.5%，化学需氧量、二氧化硫排放量分别减少12.8%、16.4%。城市水质达标率100%。工业固体废物处置利用率从95%提高到97.5%。生活污水集中处理率从61%提高到80.5%。生活垃圾无害化处理率从91.4%提高到100%。循环经济被称为“铜陵模式”。 

五年来，人民生活水平显著提高。累计投入财政资金41亿元，滚动实施民生工程219项。城镇居民人均可支配收入、农民人均纯收入分别由13266元和4614元增加到24706元和10060元。全民养老保险、医疗保障和低保标准居全省前列。每千位老人社会福利床位数由14.7张增加到20张。城镇人均成套住宅面积30平方米，农村人均钢混砖混住宅面积41平方米。城市万人公交车11标台。人均预期寿命达到77.2岁。

五年来，社会事业得到全面进步。义务教育均衡发展走在全国前列，高中教育毛入学率达到104.9%。实现全国科技进步先进市“五连冠”。城乡基本公共卫生服务实现均等化，成功创建国家卫生城市。公共文化服务体系基本建成。群众体育蓬勃开展。人口和计划生育工作保持全省先进。“四优”和谐社区达标率96%，村委会自治达标率100%。平安铜陵建设成效显著，公众安全感和群众满意度居全省前列。 

同时，我们也清醒认识到，产业结构不优、环境容量不足的问题日益突出，生态环境建设和群众的期待还有差距；一些部门效能不高、少数干部精神懈怠，不能适应转型跨越的要求。我们必须高度重视，采取切实有效措施认真加以解决。 

各位代表！过去五年的成绩，是省委、省政府和中共铜陵市委正确领导的结果，是全市人民智慧、心血和汗水的结晶。在此，我代表市人民政府，向全市人民，向人大代表、政协委员，向各民主党派、工商联、无党派人士、各人民团体，向驻铜部队、驻铜单位，向所有关心、支持、参与铜陵建设和发展的同志们、朋友们，表示崇高的敬意和衷心的感谢！ 

二、今后五年的奋斗目标 

今后五年的总体要求是：高举中国特色社会主义伟大旗帜，以邓小平理论、“三个代表”重要思想、科学发展观为指导，全面贯彻党的十八大精神，按照“五位一体”的总体布局，解放思想，勇于创新，敢于争先，善谋力行，奋力推进转型跨越，全力建设“四个之城”，继续走在全省转型发展、开放发展、创新发展、和谐发展的前列。 

主要奋斗目标是：全面完成市第九次党代会和“十二五”规划目标，经济总量省内进位次，总体效益省内再领先，居民收入全省争第一，率先实现基本公共服务均等化、教育现代化和城乡一体化，为基本实现现代化打下坚实基础。主要任务是： 

———致力建设现代产业之城。把提高经济增长的质量和效益摆在首要位置，加快产业转型升级步伐，构筑铜陵特色的现代产业体系，实现低碳发展、绿色发展、循环发展和安全发展。 

坚持工业强市战略不动摇。铜基新材料、节能环保、新能源、装备制造等战略性新兴产业集群基本形成，行业地位初步确立。铜产业主营业务收入超3千亿元，有色公司超2千亿元；百亿元以上企业超10家、10亿元以上企业超30家。 

坚持优先发展现代服务业。服务业增加值占GDP比重达到35%。成为长江流域物流节点城市、有色金属交易中心、区域性休闲购物和医疗服务中心。铜文化创意和旅游业初具规模。 

坚持以转变增长方式为战略支撑。基本淘汰落后产能。万元GDP能耗下降13%，全面完成省政府下达的减排任务。建成国家循环经济示范市、国家资源综合利用示范基地。

———致力建设创新创业之城。把科技创新作为转型跨越的核心环节，把全民创业作为富民强市的基本途径，把深化改革作为推动发展的强大动力，成为国家创新型城市和创业型城市。 

区域创新体系基本健全。全社会研发投入占GDP比重提高到 2.5 %，每万人有效发明专利拥有量达到 6.5件，高新技术产业增加值占GDP比重达到30%以上。 

“人才强市”战略有效实施。人才资源占人力资源总量的比重达到27.4%以上，高技能人才占技能劳动者比例超过27%。新增各类市场主体3.3万户以上，实现成功创业1万人。 

理顺政府和市场的关系。正确发挥政府引导作用，充分发挥市场基础作用，全面发挥企业主体作用。建立健全政府服务企业、市场配置资源、企业活力无限的科学发展机制。

———致力建设精致大气之城。全面实施精致大气之城建设“八大工程”，总投资1100亿元以上。努力建设国际铜都、山水城市、宜居家园、休闲胜地。 

基本建成生态山水家园。建成区面积100平方公里以上。主城区现代服务业进一步集聚。东部城区成为产城一体的活力新城。南部城区成为滨江文化旅游新城。跨江发展取得重大进展。 

内外交通体系更加完善。完成区域内高速铁路、高速公路建设，建成亿吨级以上现代化港口，区域交通枢纽地位进一步确立。建成城市内半小时快速交通网和快速公交体系。

城乡环境质量趋于友好。人均公共绿地13平方米以上。森林覆盖率34%以上。完成废弃矿山生态修复和水生态治理工程。基本实现农业品牌化、农村田园化、农民市民化。成为全国文明城市。 

———致力建设平安幸福之城。让人民过上更加美好的生活，是铜陵发展的根本目的。完善民生工程实施长效机制，实现“十二五”居民收入倍增计划，让发展成果更多惠及全市人民。 

率先实现基本公共服务均等化和教育现代化。全面建立城乡均等的基本公共服务。人人享有基本医疗卫生服务。每位老人享有基本养老服务。每个困难家庭都能住上保障性住房。基本实现学前教育普及化、义务教育均等化、高中教育优质化、高等教育特色化。

社会自我管理能力大幅度提升。提高城乡社区自治水平，增强城乡社区服务功能。提高社会组织的自我服务和管理能力。完善群众诉求表达、利益协调、权益保障机制，健全社会治安防控体系，形成源头治理、动态管理、应急处置相结合的社会管理机制，把铜陵建设成为最稳定、最安全、最和谐的城市之一。 

三、2013年的主要工作 

2013年，是全面贯彻落实党的十八大精神的第一年，是新一届政府的开局之年。做好今年工作，必须按照中央、省经济工作会议精神和市委九届七次全会的要求，紧紧围绕“四个之城”建设，以提高经济增长质量和效益为中心，稳中求进、好中求快，不等不靠不浮躁，抓转型上总量，抓项目增后劲，抓改革激活力，抓民生促和谐，扎实起步，开好新局。 

综合考虑，经济社会发展的主要预期目标是：生产总值增长11%以上；财政收入与生产总值增幅保持同步；全社会固定资产投资增长20%以上；社会消费品零售总额增长15%以上；居民消费价格总水平涨幅控制在3.5%左右；城乡居民人均可支配收入增长13%以上；城镇登记失业率控制在3.8%以内；完成省下达的节能减排任务。重点抓好以下工作： 

（一）着力保持经济平稳较快增长 

开展项目建设推进年活动。围绕产业发展和城乡建设，分解落实市和县区重点建设项目、明确时间节点，以强化服务促项目落地，以征迁力度促项目开工，以效能建设保项目进度，做到项目开工个数、投资规模、施工质量和建设进度“四个”超历史。实施80个以上战略性新兴产业重点项目和百项转型升级技改项目，促进PCB、光电等特色产业园集群发展。推进75个亿元以上服务业项目建设。皖中南物流甩挂中心建成运营；支持广东物资集团、卓尔集团等物流业龙头企业整合资源、打造品牌，充分发挥区位优势；引进和培育亿元以上规模商贸流通企业5个以上。确保2个以上重大项目纳入皖南国际文化旅游示范区。 

推动大中小企业更快成长。实施百亿元企业服务计划，支持有色公司成为全国最大最强的铜冶炼企业、重要的铜加工企业和有重要影响力的有色金属交易企业；支持全威铜业、精达股份、铜化集团做大做优做强；支持旋力特钢、海亮铜业、有色高导铜材、富鑫钢铁、新亚星焦化、欧宝天奇等企业向百亿企业迈进；引进一批百亿元的现代服务业项目。抓住国家大力扶持民营经济的新机遇，实施民营经济促进计划，加大对民营经济的政策支持、融资支持，深入开展千人创业扶持计划，激发全民创业热情，促进民营经济大发展。实施中小企业希望工程，促进94户省级重点调度中小企业加速成长，力争50户中小企业上规模。 

增强集聚发展要素的能力。切实落实招商选资、招大引强，以六大战略性新兴产业分配招商任务，以引进亿元以上投资项目、知名品牌企业、行业龙头企业为招商奖励对象，以项目落地实绩为招商考核标准，全年实际利用外商直接投资4亿美元，增长20%，实际利用内资670亿元，增长18%。改进政府融资平台运行机制和奖励办法，加快融资能力建设，全年平台融资不少于100亿元。鼓励金融机构来铜设立分支机构，积极改进信贷环境，全年信贷总量增长20%以上。支持企业发行企业债、中期票据、短期融资券以及中小企业集合债，积极培育企业上市资源，力争1家企业成功上市，全年直接融资额增长50%以上。积极争取建设用地指标，土地增减挂指标不低于去年，用好废弃工矿地转用指标，完善土地指标分配和供后管理机制，清理闲置土地不低于2000亩，土地出让收入不低于25亿元，落实单位用地投资强度规定，促进集约节约用地。 

（二）切实强化发展平台建设 

提升园区产业承接能力。增强各开发园区服务能力，支持开发园区以服务企业、服务项目为导向，自主设立内部机构，自主确定内部奖惩和考核办法。经济技术开发区完成投资120亿元以上，引进5家牵动性强的行业龙头企业。产业转移示范区全面完成起步区基础设施建设，确保10个以上重大产业项目开工建设。各省级开发区要形成1-2个产业集群。全面实施国家现代农业示范区规划，着力培育一批规模化的农业龙头企业。  

突出产业服务平台建设。基本建成开源金属再生产业园并通过国家验收，争取批准设立进口废七类集装箱转关和有色金属期货交割仓库。提升国家铜铅锌质检中心能力，实现铜基新材料产业检测项目全覆盖。组建安徽铜基新材料产业技术创新战略联盟，支持有色公司建立铜冶炼及加工工程研究中心、国家铜基材料及加工技术工程实验室。推动铜工艺创意、研发、展示和交易平台建设。统筹推进信息资源共享、产品设计等产业转型升级公共服务平台建设。利用好资源型城市转型等国家和省试点政策平台，成为国家循环经济试点园区、国家城市矿产基地。综合保税区申报工作取得关键进展。 
强化科技人才支撑能力。加强企业自主创新能力建设，新建省级研发机构2家以上，新增国家高新技术企业15家以上。促进政产学研合作，发挥中科院皖江产业中心、产业技术创新战略联盟在技术研发、成果转化上的作用，提升国家示范生产力促进中心和国家级科技孵化器等平台服务能力。申报成功国家农业科技园区。完善创新创业团队引进办法，建立股权激励机制，满足对紧缺人才、高端人才的需求。 

（三）全面提高城乡建设管理水平 

提升城市功能。实施中心城区畅通工程，改造长江东、西路等主干道4.8公里，建成沿河路等10条微循环道路，购置公交车辆60台，新增停车泊位1000个以上。完成5个老旧小区环境提升工程，实施长江路等沿街立面改造和杆线下地，完成淮河大道等4条主干道亮化工程，推进高速公路出入口区域环境建设。健全争创全国文明城市长效机制，提高城市精细化管理水平，开展市容市貌、运输超载、渣土泼洒等专项整治，新改建28座城市公厕，基本完成主城区污水管网建设，启动餐厨垃圾等废弃物无害化处理，城市文明程度指数测评保持先进位次。推进主城区光纤入户和重点场所无线网络覆盖，开工建设云计算数据中心，争取纳入国家“智慧城市”首批试点。 
加快新区建设。西湖新区在建项目80个以上，完成投资75亿元以上，加快西湖片区整体开发和建设，确保建成西湖公园、市博物馆、市体育中心、青铜帝国一期、金九维景大酒店等重点项目，国际铜文化创意产业园、市文化馆新馆、市民中心全面开工。启动东部城区基础设施一体化建设，顺安政务及商业片区初具规模，开工建设京福高铁铜陵北站站前广场及连接线，示范园片区安置房建设全面展开。南部城区文化旅游开发取得实质性突破，基础设施进一步完善，启动大通文化旅游项目建设。启动普济圩区域基础设施规划建设。 

强化区位优势。配合做好京福高铁暨铜陵长江公铁大桥、宁安城际铁路建设。实现铜宣高速铜陵段复工，实施沿江公路铜陵段、黄浒至白杨坡段一级路改造，朱永路、滨江大道南北段主体工程完工，建成长江东路东村至竹园段。强化港口岸线资源整治和开发，初步完成滨江区域小码头拆除，开工建设安喜物流等3个现代化港口，启动顺安河三级航道整治项目，争取南夹江整治项目通过国家审批。 

建设美好乡村。全面落实全国城镇发展改革试点任务，以城乡一体化建设为主要内容，以乡村建设为重点，提高城镇化质量。结合土地整治、土地流转和现代农业发展，整合涉农资金，完成33个美好乡村示范点建设。深入推进农村环境连片整治，实现农村清洁工程全覆盖，治理水土流失面积15平方公里。培育农村产权交易市场，深化农村产权抵押融资工作，探索建立产业发展和乡村建设、龙头企业和农民利益良性互动的体制机制。

（四）努力推进生态环境建设 

严格节能减排管理。落实能耗和污染物排放总量控制、节能和环境影响评估、节能减排目标管理责任制等制度，强化日常调度和监管。全面推行重点领域、重点企业能耗对标管理，超标企业坚决停产整改；强化合同能源管理，实施15个节能改造示范工程。实施水泥、电力企业氮氧化物治理等减排重点工程，配套完善环保设施。坚决淘汰落后产能。加快实施经济技术开发区循环化改造项目，提高产业链条耦合和资源综合利用水平。

实施重点区域环境整治。落实重金属污染治理规划，启动循环园区域重金属污染治理项目。启动横港扫把沟地区环境综合整治及产业提升实施方案，关闭搬迁污染企业，实施居民搬迁，年内完成狼尾湖环境综合整治。基本完成滨江生态岸线整治和建设，启动滨江生活岸线区域开发建设，同步整治城市集中式饮用水水源保护区，实现区域环境逐步好转。 

加大生态修复力度。实施矿山生态环境恢复规划，加快废弃矿山植被恢复、地质灾害治理项目。开展矿山开采专项环境整治行动，推进“绿色矿山”创建活动，减少粉尘排放。完成十万亩森林增长和绿化提升工程年度任务，建成铜都大道绿化带等3条生态廊道，推进大铜官山公园和东湖湿地建设。继续强力整治全城区扬尘污染，加大南部城区扬尘治理力度，可吸入颗粒物浓度再降低2个百分点以上。 

（五）大力发展民生和社会事业 

完善就业和社会保障体系。坚持民生优先，继续实施48项民生工程，财政投入增长18%以上。创建充分就业城市，开发1000个公益性岗位安置就业援助对象，扩大企业工资集体协商覆盖面。实现城乡居民医疗保险并轨运行，继续走在全国前列，今年起有色公司职工医疗保险纳入市级统筹。积极探索社会保险一卡通制度，探索事业单位养老保险制度改革。完善城乡低保、农村五保供养和孤儿保障标准动态调整机制。新开工保障性住房20000套、竣工9200套；全面完成成片棚户区改造任务，启动非成片棚户区改造；新开工安置房6652套、竣工11374套；城中村改造完成投资20亿元；改造农村危房2300户。扩大住房公积金缴存面。 

发展教育和文化事业。落实教育现代化实施方案，推进城乡教育资源均衡配置，完成16所义务教育阶段学校标准化建设，完成学前教育三年行动计划。建设33个重点村文化活动室，获得全国公共文化服务体系示范区创建资格。大力发展文化产业。积极发展新闻出版、广播影视、哲学社会科学等事业。办好第三届安徽民俗文化节。 

统筹发展各项社会事业。继续推进铜陵好人评选活动，广泛开展社会志愿服务活动。深入开展家庭医生等便民服务，支持市级医院服务能力建设。深化幸福家庭创建活动。举办市第十一届运动会暨第三届全民健身运动会，成为全国全民健身示范试点市。实施4所农村敬老院转型，养老机构床位数增长15%以上。争创全国无障碍建设城市。做好国防动员、民兵预备役和人防工作，持续开展全国双拥模范城创建活动。充分发挥工会、共青团、妇联等人民团体的重要作用，加强民族、宗教、外事、侨务、对台、气象、保密、档案、史志、红十字、防震减灾等各项工作。 

（六）深入推进和谐社会建设 

创新社会管理和服务。深化社区综合体制改革，全面推行社区兼职委员制，在7个小区开展居民自治试点，规范自治行为。加强城乡社区标准化建设，建成25个示范型农村社区服务中心，新改建2个城市社区服务用房和4个小区居民活动场所。实施社会组织市级孵化园工程，社会组织年增长率不低于7％；在教育、卫生等9个领域开展购买社会服务试点。推进事业单位分类改革，基本完成承担行政职能事业单位改革，全面完成经营性事业单位转企改制，完善公益性事业单位内部管理制度，逐步将政府部门行业管理事务性、辅助性职能转移给社会组织。 

强化平安铜陵建设。加强社会治安综合治理，开展治安突出问题和治安复杂区域专项整治，加快治安防控体系向农村延伸，严厉打击各类违法犯罪活动，不断增强人民群众安全感。深入开展“四大、四进、四送”活动，健全矛盾纠纷源头治理和化解机制，预防和妥善处置群体性事件，维护群众合法权益。完善食品药品安全监管长效机制。严格落实安全生产责任制，全面推行企业安全生产标准化管理，提高保障公共安全和应对突发事件的能力。

四、加强政府自身建设 

各位代表，经验告诉我们，政府作用发挥的好，就会成为转型跨越的重要支撑；反之，将会成为改革发展的最大约束。实现“四个之城”建设的宏伟目标，迫切需要强化服务经济社会发展的导向，迫切需要提高服务经济社会发展的能力，迫切需要增强各级政府工作人员加快发展的忧患意识、机遇意识和创新意识，迫切需要振奋各级政府工作人员的精神，建设服务高效、人民满意的政府。 

切实增强服务发展能力。进一步取消和调整审批事项。推行联合审批、网上审批。推进政务服务标准化、行政程序法定化。除涉及国家安全外，所有审批项目一律进政务服务中心办理。健全公开承诺、即接即办、首接办结等制度，审批时限一般都压缩到5个工作日以内。开展攻坚破难专项行动，在项目建设推进、征迁拆违提速、城区环境整治、市区交通畅通、治理庸懒散奢等方面制订专项行动方案，确保取得实效。政府工作人员必须深入县区和企业解决实际问题，市政府负责同志每月到每个县区（开发园区）现场办公一次，部门负责同志根据工作需要定期到县区、企业现场办公，努力打造公平公正、全国一流的发展环境。 

切实增强服务群众能力。扩大群众参与度，凡是民生事项，都交由群众作主；凡是经济社会发展的重大决策，都与群众平等沟通；凡是群众反映的现实困难诉求，都在第一时间响应；凡对群众承诺的事项，都做到项项兑现、件件落实。整合政府服务热线，实现“一个号码找政府”，打造全天候政府。落实机关干部联系困难家庭制度，为群众排忧解难。继续加大政府信息公开力度，强化网络问政的工作落实机制，及时回应百姓关切。敬畏人民就是敬畏历史，政府工作人员必须用心感受民生，尊重人民意愿，服务人民需要。 

切实增强依法行政能力。依法接受人大及其常委会的法律监督、工作监督，主动接受政协的民主监督，提高人大议案、代表建议和政协提案办理质量，充分听取各民主党派、工商联和各人民团体、无党派人士的意见，广泛接受社会公众和新闻舆论监督。深入推进规范行政执法示范市建设，全面推行权力运行公示制度，严格依照法定权限和程序行使权力，严肃落实行政执法责任。完善重大行政决策咨询、论证、听证等制度。政府工作人员必须牢固树立法治理念，带头学法、遵法、守法、用法。 

切实增强执行落实能力。牢记责任，反对空谈，崇尚实干，摒弃坐而论道，坚持实干兴铜。改进文风会风，提高行政效能，文要管用，会要议事，议后要落实。抓落实就是解决矛盾，各级各部门的落实能力是执政能力的体现，是领导能力的检验，是党性强不强的标志。抓得要紧、一抓到底，直至目标变为现实。完善岗位管理、目标管理、绩效考核、督查落实等责任机制。强化行政问责，出实招、动真格、敢碰硬，严肃查处有令不行、有禁不止、推诿扯皮和不作为、慢作为、乱作为等行为。政府工作人员必须说了干、定了办，敢作为、勇担当，言必信、行必果。 

切实增强廉洁从政能力。严格执行改进工作作风、密切联系群众的各项规定，认真贯彻党风廉政建设责任制，完善惩治和预防腐败体系，抓好廉政风险防控，深化行政权力阳光运行平台建设，完善政府招投标、政府项目投资和财政资金使用等管理制度，从源头上防止腐败滋生。坚持厉行节约，压减公用经费，降低行政成本。加强行政监察和审计监督，严肃查处违纪违法案件。政府工作人员必须公正处事，廉洁办事，树立良好形象。

各位代表！美好蓝图已经绘就，新的征程已经起航。让我们在省委、省政府和中共铜陵市委的坚强领导下，高举中国特色社会主义伟大旗帜，紧紧团结和依靠全市人民，解放思想，开拓进取，万众一心，奋力拼搏，为全面加快“四个之城”建设、谱写人民美好生活的新篇章而努力奋斗！
